# Garlic Substrate Induces Cucumber Growth Development and Decreases Fusarium Wilt through Regulation of Soil Microbial Community Structure and Diversity in Replanted Disturbed Soil

**DOI:** 10.3390/ijms21176008

**Published:** 2020-08-20

**Authors:** Ahmad Ali, Muhammad Imran Ghani, Ding Haiyan, Muhammad Iqbal, Zhihui Cheng, Zucong Cai

**Affiliations:** 1 College of Horticulture, Northwest A&F University, Yangling 712100, China; ahmadhort87@nwafu.edu.cn (A.A.); imran_pak@nwsuaf.edu.cn (M.I.G.); woaimama195710@nwsuaf.edu.cn (D.H.); 2 School of Geography Science, Nanjing Normal University, Nanjing 210023, China; zccai@njnu.edu.cn; 3 Institute of Soil Science, PMAS-Arid Agriculture University, Rawalpindi 46300, Pakistan; miqbalkhalid@gmail.com; 4 State Key Laboratory Cultivation Base of Geographical Environment Evolution 14, Nanjing 210023, China; 5 Jiangsu Provincial Key Laboratory of Materials Cycling and Pollution Control, Nanjing Normal University, Nanjing 210023, China

**Keywords:** garlic substrate, plant growth, microbial community structure, microbial interaction, cucumber yield, Fusarium incidence inhibition

## Abstract

Garlic substrate could influence plant growth through affecting soil microbiome structure. The relationship mechanism between changes in soil microbial communities, disease suppression and plant development, however, remains unclear, particularly in the degraded soil micro-ecological environment. In this study, garlic substrates as a soil amendment were incorporated with different ratios (1:100, 3:100 and 5:100 g/100 g of soil) in a replanted disturbed soil of long-term cucumber monoculture (annual double cropping system in a greenhouse). The results indicated that higher amount of C-amended garlic substrate significantly induced soil suppressiveness (35.9% greater than control (CK) against the foliar disease incidence rate. This inhibitory effect consequently improved the cucumber growth performance and fruit yield to 20% higher than the non-amended soil. Short-term garlic substrate addition modified the soil quality through an increase in soil organic matter (SOM), nutrient availability and enzymatic activities. Illumina MiSeq sequencing analysis revealed that soil bacterial and fungal communities in the garlic amendment were significantly different from the control. Species richness and diversity indices significantly increased under treated soil. The correlation-based heat map analysis suggested that soil OM, nutrient contents and biological activators were the primary drivers reshaping the microbial community structure. Furthermore, garlic substrate inhibited soil-borne pathogen taxa (*Fusarium* and *Nematoda*), and their reduced abundances, significantly affecting the crop yield. In addition, the host plant recruited certain plant-beneficial microbes due to substrate addition that could directly contribute to plant–pathogen inhibition and crop biomass production. For example, abundant *Acidobacteria*, *Ascomycota* and *Glomeromycota* taxa were significantly associated with cucumber yield promotion. *Firmicutes*, *Actinobacteria*, *Bacteroidetes*, *Basidiomycota* and *Glomeromycota* were the associated microbial taxa that possibly performed as antagonists of Fusarium wilt, with plant pathogen suppression potential in monocropped cucumber-planted soil.

## 1. Introduction

The demands for food and vegetables are increasing globally with the rapid growth of the human population. Plastic greenhouse vegetable cultivation (PGVC) has been developed worldwide, which reflects the modern trend of intensive crop production for fulfilling vegetable consumption [1]. The PGVC area has reached 3.5 million hectares globally [2] and such a type of protected cultivation offers greater economic benefits than those associated with open-field cultivation [1,3]. China is the leading producer of PGVC crops and the interest of commercial farmers, especially in the northern parts of China, has extended dynamically, where production areas, structure and crop diversity have gradually expanded under PGVC. It has been estimated that China has more than 1.7 million hectares of PGVC area and this contributes more than 80% of the total vegetable production on the mainland [4,5].

Regrettably, the global PGVC development is becoming increasingly problematic and eco-susceptible for sustainable vegetable production [6]. The conventional management system of PGVC crops has been accelerated by long-term anthropogenic activities, such as single crop repetition, the excessive use of synthetic fertilizers and hostile plant protection measures. This could profoundly affect crop productivity and the soil micro-ecological environment [7,8,9,10]. Of particular concern, traditional cucumber (*Cucumis sativus* L.) monoculture with a double-cropping system is likely to become vulnerable to soil-related obstacles that exert monoculture-related production constraints in intensive PGVC regions of northern China. Soil sickness, soil-borne pathogens and the accumulation of autotoxins/phytotoxins have been associated with cucumber continuous cropping systems [9,11,12]. Previous studies revealed long-term negative soil–plant feedback, in which a 50% reduction of the total dry biomass occurred over seven years of consecutive monocropping of cucumber [8,9,13,14]. In addition, re-cropping PGVC soil has shown 31–42% lower soil organic matter (SOM) than in open field soils [10,15] and thus degraded soil becomes vulnerable to the incidence of Fusarium wilt [13,16], caused by the soil-borne fungus *Fusarium oxysporum f.* sp. *cucumerinum*.

Thus, continuous cropping in this growing system disrupts the soil micro-ecological environment and alters the soil microbial structure, which consequently affects the aboveground plant growth [12,17]. Rhizosphere soil harbors significantly more microbial abundances of copiotrophic communities (fast-growing microbes) than bulk (root-free) soil, which substantially alter soil microbiomes [6]. Rhizosphere microbes can influence the aboveground plant biomass of the host plant through their influence on nutrient cycling, biological functions and pathogen status, thus acting as a bottom-up force [13]. Consequently, the plant-associated soil microbial shift could be an important driver of soil fertility, plant health and the functioning of terrestrial ecosystems [18]. However, the plant growth observation associated with the dynamics of soil bacterial and fungal communities in amended substrate amended cucumber-planted soil have not been deeply assessed using high-throughput molecular approaches.

Following the significance of allelopathic crops in agriculture production, their component usage on the soil and effect on plant growth offer a new dynamic choice for sustainable development by optimizing its concentration, mode of action and species adaptability. This means that garlic (*Allium sativum* L.) may be a good preceding crop, which is widely cultivated with other crops to alleviate their continuous cropping obstacles [19,20,21]. Its inherent positive allelopathic potential has attracted wider attention in recent years as a new approach to crop production [7,22]. It is noteworthy that this dominant plant species is planted as a model cropping system (garlic intercropping or garlic–crop rotation) in China to improve plant growth and physiology [23] and restructure the soil microbial/biochemical properties as compared to mono-cropping [19,20,21,24,25].

Garlic stalks/residues are a highly viable by-product of harvested garlic crops, which could be utilized as a sustainable soil amendment. Previously, in [26], garlic-mediated plant–soil feedback was observed, in which incorporated garlic substrate stimulated physiological growth development through triggering the antioxidative abiotic stress mechanism in eggplant. A high-throughput Illumina MiSeq approach further elucidated that garlic substrate amendment potentially affects the soil microbial community structure [27]). Despite that, the potential use of garlic substrate as a micro-ecological mediator of plant–microbe interactions is still limited to other cash crops such as cucumber, one of the most economically significant vegetable crops in the world. It is, however, still to be extensively investigated whether garlic substrate-induced soil–microbial changes play some role in Fusarium wilt inhibition and the enhancement of cucumber yield.

Through the current study, our effort was to improve the degraded crop-growing environment of cucumber-planted soil with short-term garlic substrate amendment. We hypothesized that (1) changes in the soil microbiota caused by garlic substrate affect cucumber growth and reduce the incidence of Fusarium wilt and (2) garlic substrate could inhibit a soil-borne pathogen fungus (*Fusarium* taxa) while stimulating its antagonists. The objectives of this study were (1) to investigate the possible role of garlic stalk amendment with different ratios on the soil quality, plant growth and physiological attributes of replanted soil, (2) to elucidate the responses of soil microbial communities to garlic stalk addition and (3) to assess the garlic-mediated plant–microbial changes to identify further which significant soil edaphic factors or microbial species are involved in cucumber yield promotion and thus decrease the incidences of cucumber Fusarium wilt.

## 2. Results

### 2.1. Effect of Garlic Substrate on Plant Growth Parameters and Fusarium Incidence Rate

The results show a significant increase in the plant height and leaf area of cucumber (*Cucumis sativus* L.) with increasing garlic substrate concentrations (Table 1). At a low concentration (1:100), the shoot fresh and dry biomass decreased slightly compared to the CK and attained its highest value at a concentration of 5:100. The highest root biomass and fruit fresh weight were observed in the T2 treatment (3:100), while the fruit length did not change significantly among the treatments. A pronounced effect on the yield was typically evident 95 days after incorporating the garlic substrate into the soil and appeared especially significant at higher concentrations (*p* < 0.05). The mean cucumber yield was increased by 20% at the highest dose applied when compared to the control plants. The cucumber *Fusarium* incidence rate (%) was significantly different among the four treatments and is shown in Table 1. A high incidence of Fusarium wilt occurred in the CK. Compared to the CK, low concentrations of garlic substrate had no obvious inhibitory effect on the disease incidence. The T1 (1:100) and T2 (3:100) treatments had an average disease incidence rate of 10.4% and 21.1%, respectively. The highest decrease in disease incidence % was observed at a higher dose of garlic substrate addition (5:100) and the inhibitory effect was 35.9% greater than in the control.

### 2.2. Effect of Garlic Substrate on Chlorophyll and Leaf Gas Exchange Parameters

**Figure 1:**
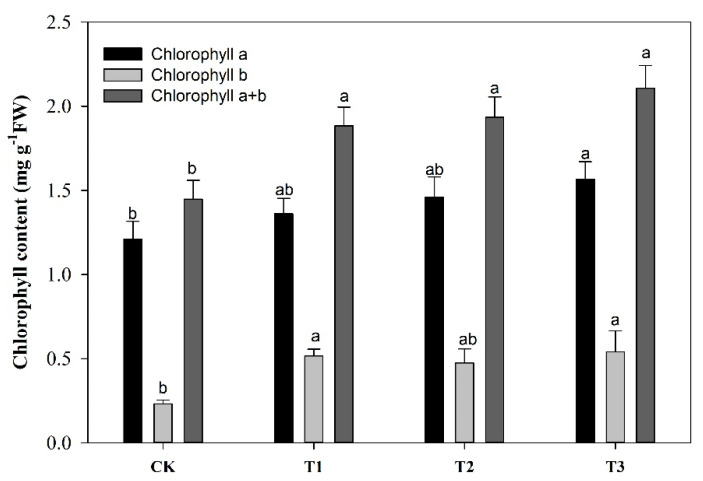
Chlorophyll a (Chl a), chlorophyll b (Chl b) and total chlorophyll contents (Chl a + b) in the leaves of cucumber under different concentrations of garlic substrate. Values represent the mean SE (*n* = 3). Different letters indicate a significant difference between treatments at *p* < 0.05, according to the LSD test.

**Figure 2:**
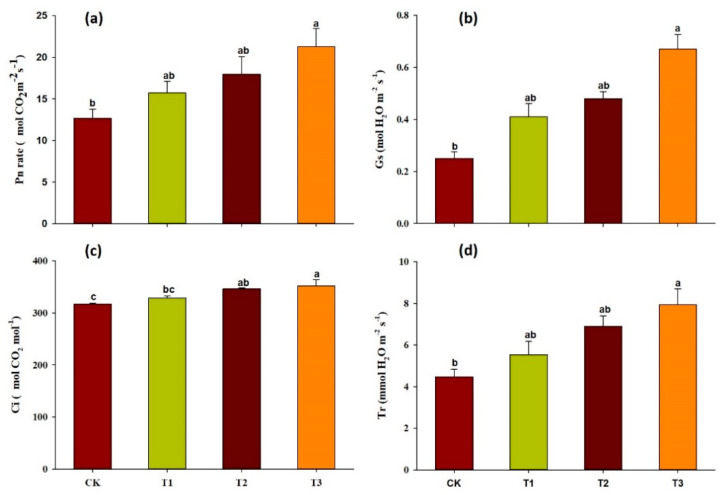
Photosynthetic gas exchange parameter analysis. Net photosynthetic assimilation (Pn) (**a**); stomatal conductance (Gs) (**b**); intercellular CO2 concentration (Ci) (**c**); and transpiration rate (Tr) (**d**) in fully expanded cucumber leaves in response to garlic substrate addition. Data are the mean ± SD from three replicates.

The addition of the garlic substrate resulted in a considerable improvement in the formation of leaf photosynthetic pigments in comparison to the control plants (Figure 1). The garlic substrate added to the soil at the ratio of 5:100 increased Chl a (29%), Chl b (133%) and total chlorophyll (45%) contents compared to the control. In addition, plants grown under different garlic substrate treatments had considerably higher leaf gas exchange rates (Figure 2). The rates of increase in these variables in response to the increasing concentration of garlic substrate differed significantly compared to the control treatment. The highest increase in the leaf gas exchange measures relative to the control treatment was observed at the garlic substrate application ratio of 5:100. The resulting changes were 67, 168, 10 and 77% for Pn, Gs, Ci and Tr, respectively.

### 2.3. Effects of Garlic Substrate on Changing the Soil Characteristics of Replanted Soil

The treatment effects on the soil chemical and biological properties are shown in Table 2. The data show the temporal changes in alkalinity depending on the magnitude of the garlic substrate addition. A low concentration (e.g., 1 g addition) induced a significant temporary increase in the soil pH, while the values decreased proportionally after the soil was treated with a higher rate of garlic substrate. The addition of garlic substrates up to a ratio of 5:100 caused a slight but significant reduction in pH, a decrease of 0.12 units, compared to the control. Similarly, the soil electrical conductivity (EC) value dropped marginally at the lowest ratio, while the addition of a higher amount (5:100) of garlic substrate significantly increased the EC value (611.5 µs·cm^−1^) compared to the control (588.5 µs·cm^−1^). The organic matter and available nutrient contents were also improved after the application of substrate amendments. However, the effect of the amendments between the incorporated treatments was variable and not of a greater magnitude in the case of N and P availability. The highest amount of treatment containing the highest amount of organic matter, 35.89 g/kg (39%), added 113.39 mg/kg (50%) and 136.61 mg/kg (87.57%) more available N and P contents in the soil, respectively, than the control. The K availability in these soils ranged from 333.18 to 355.83 mg·kg^−1^ across all the treatments, and the maximum K contents (up to 6%) were induced when garlic substrate in a 3:100 ratio was incorporated.

The addition of garlic substrate amendment had a significant effect on soil biological indicators. N cycling (urease) activities in all the amended treatments were higher than those of CK with percentage increases of 39.54%, 53.26% and 54.76% in T1, T2 and T3, respectively. The catalase and invertase activities only responded significantly (*p* < 0.05) after the soil was amended with a higher dose of garlic substrate at a ratio of 5:100, and the corresponding values increased by 25% and 44%, respectively, compared to the non-amended soil. Similarly, the P cycling (alkaline phosphatase) activity was significantly higher (3.35 mg·g^−1^) in 5% garlic-amended soil, followed by the other treatments over a short-term period (95 days).

### 2.4. Composition of Bacterial and Fungal Communities as Influenced by Garlic Substrate

**Figure 3:**
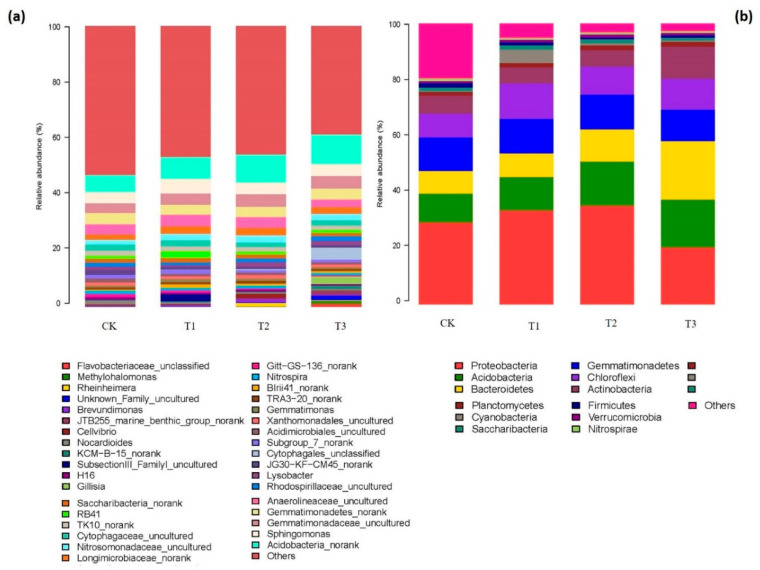
(**a**) Relative abundances (%) of bacterial community composition at the genus level. (**b**) Relative abundances of community proportions of bacteria samples at the phylum level under different treatments.

After processing the raw sequence reads by QIIME Pipeline through the Illumina MiSeq platform, we detected a total of 922,295 effective sequences, with an average length of 300–450 bp across all the treatment samples. A total of 458,807 and 463,488 high-quality sequences were identified for the bacterial 16S gene and fungal 18S genes, which represented >49% and 50% of the total, respectively. All sequences were classified from phylum to genus, and the relative abundance (RA) of the bacteria at the phylum and genus levels among all the treatments are shown in Figure 3. *Proteobacteria, Acidobacteria, Bacteroidetes, Gemmatimonadetes, Chloroflexi, Actinobacteria, Planctomycetes* and *Cyanobacteria* were the eight most dominant phyla, accounting for 77–92% of the total bacterial 16S rRNA gene sequences across all of the treatment samples. A phylogenetic analysis indicated that *Proteobacteria* was the predominant phylum in all the samples, and it gradually increased by 14.68% and 20.65% in the garlic-amended treatments of T1 and T2, respectively, while it declined moderately by 30.58% with the addition of the garlic substrate in T3. Other dominant phyla, such as higher abundances of *Acidobacteria* (15.25–65.51%) and *Bacteroidetes* (4.75–15.77%), were found in the amended treatments compared to the control, and the RAs of these phyla increased considerably with the increasing garlic substrate dosage (Figure 3b). A lower proportion of garlic substrate with a ratio of 1% (1:100) and 3% (3:100) caused a slight reduction in the *Actinobacteria* abundance, and a 78.34% increase was recorded at the highest ratio of garlic substrate added. Similarly, the phylum *Chloroflexi*, with an abundance of 11.2% in the control, increased by 49.60, 16.94 and 30.07% in T1, T2 and T3, respectively. Additionally, no significant changes were detected in the *Gemmatimonadetes* phylum across the treatments.

Using all the genera that could be identified, different genera or groups were selected as representative genera for the community comparison between the treatments based on the genus abundance in all the samples (above 0.1%). As shown in Figure 3a, the top five abundant genera or groups, *Acidobacteria_norank*, *Sphingomonas*, *Gemmatimonadaceae_uncultured*, *Gemmatimonadetes_norank* and *Anaerolineaceae_uncultured*, were selected based on the comparison requirements. Consequently, the RAs of these bacterial genera also varied in different garlic substrate treatments. In particular, *Acidobacteria* was frequently at the top of the genus-level distributions, and its RA increased with increasing concentrations of garlic substrate.

**Figure 4:**
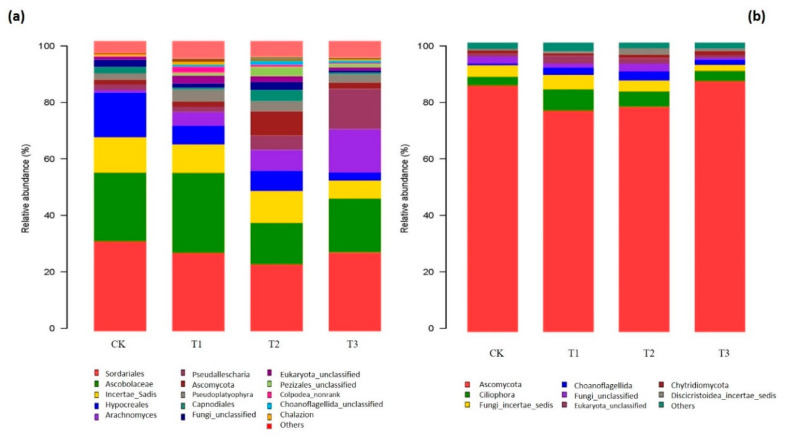
(**a**) Relative abundances of fungal community composition at the genus level. (**b**) Relative abundances of community proportions of fungal samples at the phylum level under different treatments.

The different taxonomic complexity (phyla and genera) of the fungal community is visualized on the basis of the RAs in Figure 4. Short-term soil amendment with garlic substrate apparently imposed a differential impact on the fungal community structure. The dominant fungal phyla across all soil samples were *Ascomycota*, *Ciliophora* and *Fungi_incertae_sedis*, followed by varying occurrences of *Choanoflagellida*, *Fungi_unclassified*, *Eukaryota_unclassified* and *Chytridiomycota* (Figure 4b). Ascomycota abundance substantially decreased in the garlic-amended soil with a lower concentration of T1 (10.14%) and T2 (8.56%) and slightly increased (1.82%) at higher concentrations. The total *Ciliophora* (14.23–132.01%) and *Choanoflagellida* (202.28–456.63%) abundances were significantly higher in the three soil amendments (1%, 3% and 5% of raw garlic substrate RGS) compared to the control. The majority of the prevailing fungal genera of all the soil samples were *Sordariales_norank*, *Ascobolaceae_uncultured*, *Incertae_Sedis_incertae_sedis* and *Hypocreales_unclassified*, which declined in the amended soil, while *Arachnomyces*, *Pseudallescheria*, *Ascomycota_unclassified* and *Pseudoplatyophyra* were enriched in the amended soil (Figure 4a). The rest of the other genera appeared to have few significant changes under all the treatments.

### 2.5. Changes in Soil Bacterial and Fungal Alpha Diversity

Sequence clustering revealed that the alpha diversity indices of both the 16S rRNA and 18S rRNA sequences significantly affected after garlic substrate amendment at 0.03 of the genetic distance, as shown in Table 3. The maximum number of operational taxonomic units (OTUs) (2268 bacteria, 141.7 fungi) was found in the T2 treatment compared to CK (2190 bacteria and 110.9 fungi, respectively). The Shannon–Wiener diversity indices of the bacteria and fungi showed similar trends and they increased with significant differences (*P* < 0.05) in the T2 treatment, which indicates a high degree of bacterial and fungal diversity. The highest values of the estimated richness (ACE and Chao1) were recorded under T2 for bacteria and T1 for fungi, respectively, compared to the control, suggesting that the species richness and diversity of bacterial and fungal communities in the soil were affected differently in the garlic stalk-amended soil.

### 2.6. Differences in Community Composition and Diversity

The rarefaction analyses of the OTU richness between different libraries for bacteria and fungi detected in all the soil samples (Supplementary Figure S1). Most rarefaction curves reached plateaus after ~20,000 pyrotags were sequenced, demonstrating that the sampling effort covered almost the full extent of the taxonomic diversities of the samples using a genetic distance of 3%. The pattern of bacterial OTU richness was relatively stable, whereas fungal OTU richness was diverse across all samples. The community overlaps are illustrated in the Venn diagrams, showing that the overall garlic-amended soils with different concentrations exhibited a greater number of unique OTUs (T3: 48 for bacteria, T1: 18 for fungi) than CK (38 for bacteria, six for fungi), respectively (Supplementary Figure S2). The highest number of shared bacterial OTUs was 2776 for the intersection between T2–T3, and 167 were detected for those pairwise treatments of T1–T2 for shared fungal OTUs.

**Figure 5:**
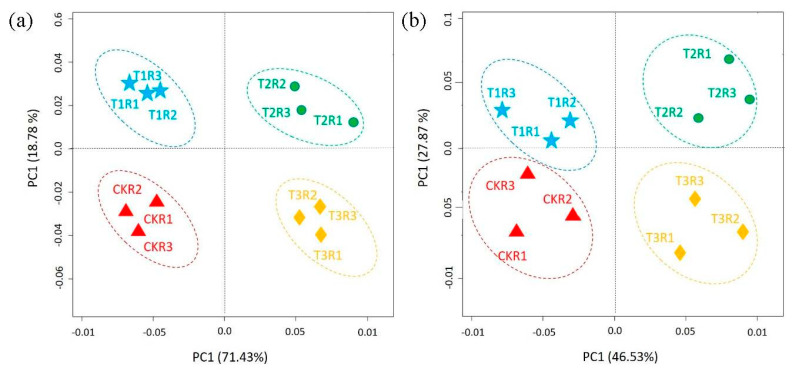
Principal component analysis of the bacteria (**a**) and fungi (**b**), revealing the community differences among all the soil samples.

To get a better insight into the differences of the soil microbial community, we applied principal component analysis (PCA) and observed the general difference in the composition of bacterial and fungal communities among the different samples. The four soil samples were distributed separately at 71.43% and 18.78% on the PCA vector 1 and 2 axes for the bacterial community (Figure 5a). Likewise, the PCA variation (46.53% for PC1 and 27.87% for PC2) accounted for the fungal community across all samples (Figure 5b). All the soil samples were distinct from the others, indicating the large community differences under different conditions.

### 2.7. Relationship among Key Soil Properties, Crop Yield, Disease Incidence and Microbial Structure

**Figure 6:**
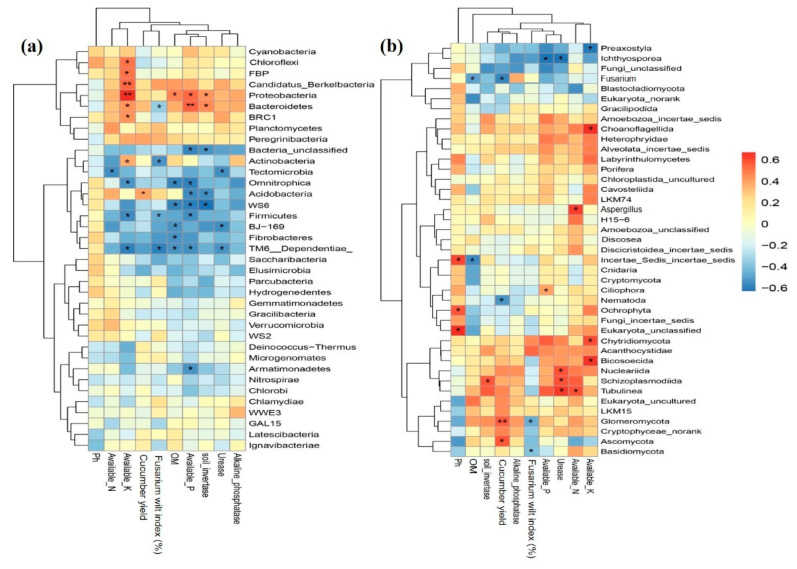
The heat map depicts the relationship of key soil edaphic factors, cucumber yield and Fusarium wilt incidence rate % with top bacterial (**a**) and fungal taxa (**b**). Soil samples were clustered using an unweighted pair group method with arithmetic mean (UPGMA) dendrogram based on Bray–Curtis similarities. The color legend and scale are provided in the figure (*blue* means low relative abundance with negative association while *yellow* and *red* mean higher abundance with positive correlation). Shaded values indicate statistically significant (* *p* < 0.05; ** *p* < 0.01) associations with correlation.

The key soil edaphic factors, such as OM content (** 0.01), invertase (* 0.05) and alkaline phosphatase (* 0.05), significantly positively correlated with cucumber yield. Fusarium wilt incidence rate negatively correlated with OM (* 0.05) and thus cucumber yield (* 0.05) (Supplementary Table S1). Heat map-based correlation analysis revealed that the bacterial phyla *Proteobacteria* and *Bacteroidetes* were present at the highest relative abundance compared to the other phyla and were significantly influenced by the soil invertase (* *p* < 0.05), available phosphorous (* 0.05; ** 0.01), available K (* 0.05; ** 0.01) and OM (* 0.05), as shown in Figure 6a. The cucumber yield was significantly positively correlated (* 0.05) with *Acidobacteria* phyla. The beneficial bacterial phyla that negatively correlated with Fusarium wilt incidence rate (%) were *Firmicutes* (* 0.05), *Actinobacteria* (* 0.05) and *Bacteroidetes* (* 0.05). The bacterial alpha diversity also affected crop yield and disease incidence rate. The observed community richness indexes (OTUs, ACE and Chao1), along with community diversity index (Shannon), were positively correlated with cucumber yield and negatively correlated with Fusarium incidence (Supplementary Table S2).

To reveal the fungal relationship, the heat map graphically shows that the abundance of the certain fungal phyla was significantly affected by the soil edaphic factors. The two abundant fungal phyla known as *Chytridiomycota* and *Chaonoflagellida* were positively correlated with the available K (* *p* < 0.05) (Figure 6b). The phyla *Aspergillus* had more reads and the factor significantly modeling this fungal abundance was the available soil N (* *p* < 0.05). The available soil P positively correlated with *Ciliophora* (* 0.05). The phyla *Incertae_sedis* also positively and negatively correlated with OM and pH, respectively. The activity of urease was found to be the dynamic factor that was positively associated with the community distribution of the fungal *Nucleariidia, Schizoplasmodilida* and *Tubulinea* taxa (Figure 6b). *Ochrophyta* was positively associated with soil pH (* 0.05). Cucumber yield was significantly positively correlated with *Ascomycota* (* 0.05) and *Glomeromycota* (** 0.01). The pathogenic *Fusarium* taxa, along with *Nematoda* phyla, were negatively associated with cucumber yield (* 0.05). The Fusarium wilt incidence rate was significantly negatively correlated with *Basidiomycota* (* 0.05) and *Glomeromycota* (* 0.05). The potentially pathogenic *Fusarium* taxa showed low relative abundance after garlic substrate addition and the soil OM was significantly negatively correlated with the decline of *Fusarium* taxa. In addition, the soil fungal diversity index (Shannon) negatively correlated with Fusarium incidence rate (Supplementary Table S2).

## 3. Discussion

### 3.1. Garlic Substrate Addition Improved Cucumber Growth and Reduced Fusarium Wilt Incidence %

The improvement of anthropogenic stressed soil conditions of PGVC after garlic substrate addition resulted in better cucumber growth and physiological development. The significant plant growth observations at a higher concentration of applied garlic substrate were possibly due to increased nutrient bioavailability [28]. The fresh weight of the aboveground and belowground biomass also increased as compared to the control; this could be attributed to an increase in better root growth and maximal root expansion, resulting in a greater root surface area, which enabled the plants to access more nutrients from the soil and ultimately favored plant growth [29]. Crop residue management, along with partial fertilizer applications, can serve as a vital source to improve crop production and restore essential nutrients depleted due to continuous cropping obstacles [13]. Phenological developments and field crop productivity were also reported to be increased to 50–60% under intensive cropping systems [30]. The cucumber yield in our study also increased after garlic substrate addition, and this is in accordance with the other findings which reported that yield biomass attributes are strongly associated with the improvement of the degraded soil conditions [31,32]. The disease incidence rate of cucumber Fusarium wilt also significantly decreased after garlic substrate addition and the biocontrol activity of vascular wilt pathogens could be related to root exudation function and organic compounds [33,34].

The chlorophyll pigments reflect the plant’s photosynthetic assimilation ability and determine its photosynthetic rate. The contents of chlorophyll a and b and total chlorophyll improved significantly compared to the control, which was consistent with previous assertions [22,34]. The stomatal aperture, as well as conductance, regulates the intercellular CO_2_ uptake and H_2_O_2_ evaporation in response to eco and biochemical factors. In this study, the stomatal conductance (gs) increased, which regulates gas exchange (CO_2_ and water) and allows plants to increase their CO_2_ supply under well-watered growth conditions and further determines Pn behavior. In principle, a higher gs can also be partly responsible for the corresponding increase of the leaf photosynthetic rate that is co-dominantly correlated with the high-yielding capacity of higher plants [35].

Plant growth metabolism and physiological development may become sensitive to phytotoxicity if plant materials are derived from allelopathic sources. Such residual decomposition for an extended period may lead to the accumulation of phytotoxic substances and their residual effect, causing pronounced physiological alteration at various plant growth stages [36,37]. Their allelopathic regulation (positivity) or allelopathic interferences (negativity), however, could be influenced by the residue type, amount, method and duration of incorporation and species sensitivity [37]. Unfortunately, such allelopathic interaction via crop residue addition has not been dynamically reported. To correlate with our previous study [6], we infer that, during decomposition in the vicinity, the garlic substrate did not exert allelotoxic or deleterious effects on cucumber growth and physiology, because its planted organs or biomass inputs possess different allelopathic potential depending on their mode of action [26]. Phytotoxic activity from various garlic tissues, such as root exudates and bulb extract/root extracts, have been verified to have a strong allelopathic potential and they produced a greater adverse and dose-dependent effect on receiver crops [22,34]. Based on the results, we infer that the composition of the plant materials used in our study revealed differential allelopathic behavior. The incorporation of the aboveground components as a garlic substrate had less of an allelopathic impact or produced a positive influence on cucumber growth and productivity after a short duration of decomposition.

### 3.2. Garlic Substrate Addition Changed the Soil Quality Environment

In this study, the addition of the garlic substrate potentially affected the soil chemical and biochemical properties through organic C inputs and enhanced biological activities. Initially, the soil pH increased at a low concentration, and a decrease of up to 0.12 units occurred at a higher concentration compared to the control. This reflects the sensitivity of soil pH in response to the organic amendments, which was likely due in part to the low buffering capacity [38]. The temporal changes in alkalinity after the garlic substrate addition reflect a consistent effect, as a decrease in the pH of alkaline soils has been reported in soil–plant interactions following the addition of cover crop residues [13,39]. Electrical conductivity increased when the proportion of garlic substrate exceeded 3:100 and 5:100 in soil, this could be due to the higher EC value of the garlic residue (EC 671 µS·cm^−1^) in relation to the soil (EC 582 µS·cm^−1^) (Supplementary Table S1). The increase in the soil EC after the addition of garlic substrate indicates the presence of soluble salts other than nitrates in the plant material used as amendments that were in appropriate amounts and could be useful to improve plant growth and quality traits [40].

Generally, the incorporation of C-rich crop residues and inorganic fertilizer presumably served as a putative source to retain organic carbon stocks and N mineralization. Newly added crop residues to soil are the most readily available sources of organic matter, and the pattern of nutrient release from crop residues and its cycling have an optimistic influence on plant growth and crop yield and can reduce the need for external inputs [41,42]. Our data confirm that a higher amount of incorporated garlic substrate significantly amplified the organic content (Table 2). The physical availability of organic substrates in extensively manipulated soil could rejuvenate the long-term deficiency of the OM [43,44]. Moreover, crop residue had pronounced and variable effects on the soil C content with time, and this may lead to a more significant net N mineralization [44]. Our results indicate that the incorporation of garlic stalks could exert a positive influence on nutrient cycling and the available nutrient content (NPK). In [45], it was reported that incorporated crop residues decomposed faster, resulting in the increased availability of exogenous N for microbial breakdown. Higher phosphorous (P) availability was also attributed to the addition of the residues, which could be related to the release of organic acids during decomposition or may have been due to the solubilization of the inherent garlic substrate P, resulting in the release of P [46]. K availability, however, did not consist of higher substrate concentration, and management factors or soil conditions could influence the K content as reported by [47].

Garlic substrate addition may indicate better nutrient release capacity and biological activity. It has been reported that the chemical composition of the plant tissue, rate of decomposition and microbially mediated plant residue decomposition could play an important role in the mineral-associated SOM fraction [48,49]. The aboveground plant biomass (straw, stalk, leaves, shoots) is believed to be more rapidly decomposed and ultimately accelerated the microbial degradation due to its high water-soluble carbohydrate contents and low lignin concentration, which contributed more organic C and N [50]. The results obtained in this study are in agreement with the findings of others who reported that a high dose of pepper residue addition caused greater decomposability and easily released the nutrient contents in loamy calcareous soil [51]. The N addition to our soil may generally accelerate the decomposition rate and improve the crop residue quality by regulating the high C:N ratio [52].

The effects of applied garlic stalks elucidate the biological significance in the tested soil conditions. The tendency of selected enzymatic activities to proliferate increases according to the amount of crop residues applied in extensive agroecosystems [53,54,55]. This improvement may have been attributable to the addition of OM, the stimulation of microbial growth due to increased resource availability and changes in the microbial community composition [56]. In addition, [57] described that crop productivity was positively linked by the action of biological activators that induced microbial activity. Our results suggest that the increase in urease and phosphatase activities is an excellent proxy of N and P availability. We can conclude that the addition of garlic can cause significant changes in biological activity, and strong N and P mineralization was observed in garlic-amended soils that caused yield enhancement attributes [56].

### 3.3. Garlic Substrate Addition Alters Soil Microbial Community Composition and Diversity

The Illumina MiSeq analyses revealed quantitative insights into the cluster of microbial populations in garlic substrate amendments, and microbiota composition was significantly affected, as shown by PCA analysis in this study. The most dominant bacterial phyla found in all the samples were due to the characteristics of the organic amendments, and their abundance based on the 16S rRNA gene clones increased in residue-treated soils, as reported by [58]. In particular, *Proteobacteria* and *Acidobacteria* phyla in this study have been suggested as primary decomposers of residue degradation and thus contribute to nitrogenous organic substrate mineralization [59]. The higher response of *Bacteroidetes* in the treatments is likely to have partially affected the soil quality through the mineralization of organic substrates and the cycling of essential micro- or macro-nutrients using a variety of enzymes [58]. Incorporating a higher amount of garlic substrate in the soil caused significantly higher RAs of *Actinobacteria*, which could suggest a higher degradation rate of recalcitrant organic materials and SOM and subsequently a higher organic C turnover [57,59]. The higher abundance of *Gemmatimonadetes* and *Chloroflexi* could also improve the soil condition by degrading a resistant fraction of the plant residues in the later stages of decomposition due to limited substrate availability, which is consistent with the results of [58,60]. In this study, the most abundant isolated genera, primarily *Acidobacteria_norank*, *Sphingomonas*, *Gemmatimonadaceae_uncultured*, *Gemmatimonadetes_norank* and *Anaerolineaceae_uncultured*, were significantly affected by the garlic substrate treatments (Figure 3a). Their enriched bacterial interactions in the host–plant rhizosphere have been suggested to induce changes in plant metabolism and root C exudation [61]. Soil microbial communities are primarily limited by C-deficient environments, especially in exhaustive cropping systems, and the C-amended garlic substrate could result in the proliferation of microbial growth to provide abundant carbon resources. The incorporated garlic substrate in cucumber-planted soil may increase the C-rich substrate availability for copiotrophic microbes, which could also be ecologically important to mediate microbial processes, resulting in the maintenance or improvement of the microbial taxonomic and functional diversity [6]. Therefore, the greater quantity of a dominant microbial group of *Acidobacteria*, as associated with the ratio of garlic substrate, may also be due to the consumption of surplus carbon sources. In addition, these top identified genera were prevalent and beneficial to plants by providing bio-control agents against diseases and occurring in a variety of environments [60].

Soil fungi are also an important component of microbiota, and the rhizosphere fungal community and diversity are closely linked to soil–plant health through triggering three leading functional roles, including (1) decomposing organic matter, (2) ecosystem regulators and (3) biological controllers [13,16]. The fungal taxonomic composition at the genus level also changed between cucumber-planted soil and garlic-amended soil. The genera *Capnodiales* (Ascomycota phylum) grows quickly at the flowering stage and utilizes carbon resources immediately through the rooting system. The dominant *Sordarials*, *Arachnomyces* and *Pseudallescheria* genera were observed under garlic substrate addition (Figure 4a). Certain affiliated species are the key contributors to the degradation of the high lignin content or cellulose during root-surrounding decomposition [49]. The frequent occurrence of specific fungal decomposers, such as *Ascomycota* and *Ciliophora*, reflect the nutrient-rich conditions in our planting system. Their fungal abundance may be related to the different responses of the microbes to root exudates or rhizodeposition. In particular, the dominant and populated fungal phylum of *Ascomycota* in our soil samples was probably enriched by relatively high nitrogen contents in long-term continuous cropping of cucumber cultivation. Certain *Ascomycetes* phyla have been reported as a shred of evidence for primary soil fungal decomposers in the breakdown of organic compounds [62]. The species richness and abundance of soil protists (ciliates) in this study have been suggested as useful bio-indicators of soil structure formation due to the release of phytohormones and C- and N-rich exudates [63]. This pattern reflects the inconsistent trend observed by [64], who identified identical fungal genera with the lowest abundance during straw residue decomposition. This inconsistency might be due to the difference in plant species, interactions with mutable physicochemical parameters of the experimental soil and the chemistry of organic material used in this study.

### 3.4. Relationship Mechanism of Cucumber Yield, Fusarium Wilt Inhibition Rate and Microbial Structure

Recent studies revealed that changes in microbial communities could influence plant performance and crop health [6,26,27]. Key soil environmental factors and microbial species could be a major determinant of positive feedback effects during soil–plant–microbe interactions [18]. Our results indicated that garlic-mediated cucumber yield promotion and the reduction in the incidence of cucumber Fusarium wilt was also affected certain soil environmental indicators and microbial characteristics (Supplementary Tables S1 and S2). The correlation analysis demonstrated the soil OM, nutrient contents and biological activators were the primary drivers and consequently reshaped the microbial community structure. This was partially equivalent to the findings of other researchers, who observed this trend during different organic and inorganic management practices [57,60,65]. In particular, higher richness and diversity of *Acidobacteria*, *Ascomycota* and *Glomeromycota* were uniquely correlated to the promotion of cucumber yield. This was in parallel with our previous study, which showed that garlic substrate increased the relative abundances of several microbial taxa, exhibiting plant growth-promoting or pathogen-inhibiting potentials in degraded soil environment [6,27]. Moreover, garlic substrate decreased the relative abundances of microbial taxa containing potential plant pathogens, such as *Fusarium* and *Nematoda*. Both are the causal organisms of soil-borne pathogens that induce cucumber *Fusarium wilt* and *root-knot nematodes*, respectively. The cucumber yield was negatively correlated with both taxa, inferring that higher fruit yields were associated with lower pathogen accumulations. The inhibitory effect was consistent with previous studies and showed that different garlic components changed the composition of root exudates from cucumber, which reduced disease incidence through enhancing plant resistance to pathogen infection [33,34]. Garlic substrates also induced a certain level of beneficial microbial diversity that may have a suppressing potential to control the Fusarium wilt incidence rate in our cucumber-planted soil. *Firmicutes*, *Actinobacteria*, *Bacteroidetes*, *Basidiomycota* and *Glomeromycota* were the main antagonistic microbes that were significantly associated with the decreasing cucumber Fusarium wilt incidence rate. These results indicated that garlic substrates might have exerted positive feedback effects on cucumber yield and resistance through inhibiting plant pathogens and fostering plant-beneficial microbes. As reported in previous studies, various bioactive compounds, such as thiosulfinates and disulfides produced through garlic root exudations, may act as putative biocontrol agents for preventing Fusarium wilt disease in cucumber [34,66].

## 4. Materials and Methods

### 4.1. Site Description and Garlic Substrate Used

A pot study was conducted in spring 2016 under greenhouse conditions located at the Horticulture Experimental Station (34°17′ N, 108°04′ E) of the College of Horticulture, Northwest A&F University (NWSUAF), Yangling, Shaanxi Province. This province is one of the preeminent regions for intensive commercial PGVC in China. The northwest regions have abundant garlic residue in the fields and postharvest garlic waste materials were collected from local garlic production fields in the surrounding areas and were divided into different portions. Aboveground biomass of the garlic crop, including stalks and leaves, was selected to incorporate as a plant substrate into the soil. The collection and preparation of garlic substrate was the same as reported previously [6,26]. In brief, the garlic residue was first air dried under natural field conditions and homogenized (2 mm sieves), and then stored in dry conditions prior to use as a soil amendment in the pot trial.

The greenhouse soil of this experiment was brown loamy, alkaline and classified as Eum-Orthic Anthrosol [13,67], which was collected from the top layer (0–20 cm) of cucumber-planted PGVC soil, with a 7-year history of continuous mono-cropping (annual double cropping: spring, March to June; autumn, August to October). The cultivated soil was characterized as degraded replanted soil, caused by anthropic inputs [6,13]. The basic properties of the replanted soil and garlic substrate were investigated [6] and are summarized in Supplementary Table S3.

### 4.2. Experimental Set-Up

The experiment had a completely randomized design with three replications that had the following treatments: T1: 1.0 g garlic substrate + 99.0 g soil (1:100); T2: 3.0 g substrate + 97.0 g soil (3:100) and T3: 5.0 g garlic substrate + 95.0 g soil (5:100). Soil without the addition of garlic substrate served as the control (CK 0:100). The dehydrated and finely crushed garlic substrate was thoroughly mixed and then incorporated in the soil as an amendment with different ratios. The proposed concentrations were suggested previously [6,26,68]. Each plastic pot (15 cm × 15 cm × 15 cm) contained 8 kg of replanted soil, and ten pots were used for each treatment. 

The cucumber seeds (Jinlu No.3, a commercial cultivar of this region) were obtained from the College of Horticulture, NWSUAF, China. The seeds were initially surface sterilized with 2.5% NaClO solution for 10 min, washed thoroughly with distilled water, and germinated at 28 °C in darkness. The uniformly sized seedlings were selected and transplanted into each pot (amended and non-amended), maintaining one cucumber plant per pot. Before the cucumbers were transplanted, a dose of 15 g of compound fertilizer (N-P_2_O_5_-K_2_O: 18-18-18) was added to the pots as a basal fertilization, following the local farming practices. After amendment incorporation, the soil in each pot was moistened to about 65–70% water holding capacity (WHC) by adding tap water. The water loss from each pot was checked every day and maintained at 70% WHC on a daily basis up to cucumber harvesting.

All the treatments were retained equally for 95 days of cucumber cultivation and maintained under controlled greenhouse conditions with an average temperature of 25/16 °C day/night, 70–75% relative humidity and a 750 μmoL m^−2^ S^−1^ photosynthetic photon flux density (PPFD).

### 4.3. Soil Sampling and Analyses

The soil samples from each pot were collected immediately after harvesting the cucumber plants after 95 days of cultivation. The soil sampling procedure for determining the basic soil characteristics and microbial analysis was done as previously followed by [6]. In brief, fresh soil samples (12 replicates) were directly transported to the laboratory on ice, sieved by a 2-mm mesh sieve and divided into two parts. One part of the air-dried soil samples was kept at 4 °C to analyze the soil characteristics, and another subsample was placed in a 50-mL centrifuge tube and stored at −80 °C for DNA extraction.

The soil pH (soil: H_2_O ratio 1:2.5) and electrical conductivity (soil: H_2_O ratio 1:5) were determined using a pH meter (PHS-3CU, Shanghai, China) and a conductivity meter (DDS-307, Shanghai, China), respectively. The soil organic carbon (TOC) was analyzed using the dichromate oxidation method [69]. Total nitrogen (TN) was determined using a macro Kjeldahl digestion apparatus [70]. The available P and K were analyzed using Olsen’s and flame photometric techniques, respectively [71].

### 4.4. Soil Enzyme Assays

Four soil enzymatic activities were quantified in triplicate by using colorimetric methods, as previously described [72]. Urease and invertase were assayed based on the amount of NH_4_^+^-N and glucose released through a substrate solution of 10% urea and 8% sucrose, respectively, after incubation (24 h/37 °C). The alkaline phosphatase activity was measured as a product of phenol after the use of disodium phenyl phosphate as a substrate [73]. Catalase activity was determined by using H_2_O_2_ (3.5%) as a substrate, and the suspension was titrated with 0.1 moL L^−1^ KMnO_4_ solution after shaking for 20 min.

### 4.5. Plant Morphological and Physiological Measurements

The plant growth measurements were recorded before and after the harvesting of 95-day-old cucumbers. The height of cucumber plants was measured using a measuring tape. The leaf area index was recorded for fully expanded leaves before flowering and after flowering stages by using a hand-held leaf area meter (AM-350, Hertsmere, UK). The measurements related to leaf chlorophyll contents and photosynthetic gas exchange parameter analysis were examined before the flowering stage [6]. The chlorophyll contents in 1 g of fresh tissue were extracted with 80% acetone overnight for 24 h, and the extracts of pigments *a* and *b* were determined spectrophotometrically (UV-3802, UNICO, NG, USA) at 663 nm and 645 nm [74]. Gas exchange measurements were recorded for the second fully expanded leaf from the apex by using a portable photosynthetic system (LI-6400, LI-COR Inc., Lincoln, NE, USA). The Fusarium wilt incidence rates of the plants were measured at both flowering and fruiting stages, and visual inspection of the whole plant (leaves, stem) was carried out and recorded until the harvest of the cucumber plants. The disease incidence was calculated as the percentage of infected plants in each treatment and was evaluated when the disease emerged (>20% of leaves wilted). Shoot fresh and dry weight were recorded after the harvest of cucumber. The cucumber yield (immature marketable size fruits) were handpicked regularly and measured for fresh weight.

### 4.6. DNA Extraction and PCR Amplification of 16S rRNA and 18S rRNA

The microbial DNA was extracted from 0.5 g frozen soil samples using a modified E.Z.N.A^®®^ Soil DNA Kit (Omega Bio-Tek, Norcross, GA, USA) according to the manufacturer’s instructions. The integrity and quality of the DNA extracted were confirmed using 1% agarose gel electrophoresis, and the concentration and purification were assessed using a Nanodrop ND-2000 UV–Vis spectrophotometer (Thermo Scientific, Wilmington, WA, USA). The total quantified and purified DNA was stored at −25 °C for the subsequent analyses.

The bacterial 16S rRNA gene and fungal 18S rRNA genes were amplified in triplicate by using a thermocycler PCR system (GeneAmp 9700, ABI, Carlsbad, CA, USA). The PCR primer sets (338F/806R) were used to target the V3-V4 hyper variable regions of the bacterial 16S rRNA genes [61], and its barcoded fusion forward primer was 338F (5′-ACTCCTACGGGAGGCAGCAG-3′), and the reverse primer was 806R (5′-GGACTACHVGGGTWTCTAAT-3′). The fungal primer set SSU0817F/1196R with the forward primer SSU0817F (5′-TTAGCATGGAATAATRRAATAGGA-3′) and the reverse primer 1196R (5′-TCTGGACCTGGTGAGTTTCC-3′) was used to amplify the fungal 18S rRNA genes [61]. PCR assays were performed in triplicate, and the reaction mixture (20 μL) contained 4 μL of 5 × FastPfu Buffer (Takara, Dalian, China), 2 μL of 2.5 mM dNTPs, 0.8 μL of each primer (5 μM), 0.4 μL of Fast Pfu Polymerase (Takara) and 10 ng of template DNA. The PCR reactions were initiated using the following amplification conditions: 3 min of initial denaturation at 95 °C, 27 cycles of 30 s at 95 °C, 30 s to anneal at 55 °C, and 45 s to elongate at 72 °C, and a final extension at 72 °C for 10 min. The resulting PCR products were extracted from a 2% agarose gel and purified further using an AxyPrep DNA Gel Extraction Kit (Axygen Biosciences, Union City, CA, USA) and quantified using QuantiFluor™-ST (Promega, Madison, WI, USA) according to the manufacturer’s instructions.

### 4.7. Sequence Processing and Analysis

Purified amplicons were pooled in equimolar concentrations and paired-end sequenced (2 × 300) on an Illumina MiSeq platform (Illumina, San Diego, CA, USA) according to the standard protocols by the Majorbio Bio-Pharm Technology Co. Ltd. (Shanghai, China). The raw sequence data generated in this study were deposited in NCBI with the accession number of PRJNA655356. Forward and reverse sequences were merged by overlapping paired-end reads using FLASH (V1.2.7, http://ccb.jhu.edu/software/FLASH/). All sequence reads with the same tag were assigned to the same sample according to the unique barcodes (raw tags). The raw tags were strictly filtered, and the quality of the clean tags was detected by QIIME Pipeline (V1.9.1, http://qiime.org/index.html). Briefly, ambiguous sequences shorter than 200 bp and those with an average quality score of less than 20 were identified and removed using the UCHIME algorithm (http://www.drive5.com/usearch/manual/uchime algo.html). The effective sequences of both bacteria and fungi were then clustered into operational taxonomic units (OTUs) at 97% sequence similarity using the UPARSE-OTU and UPARSE-OUT REF algorithms of the UPARSE software package (Uparse v7.0.1001, http://drive5.com/uparse/). Finally, the RDP classifier (http://rdp.cme.msu.edu/) was used to assign a representative sequence to the microbial taxa against Silva (SSU123) using a confidence threshold of 70%.

### 4.8. Statistical Analysis

The data corresponding to plant growth, soil biochemical properties and alpha microbial diversity under different treatments were subjected to a one-way analysis of variance (ANOVA) followed by an LSD test (*p* < 0.05) using the SPSS package (version 16.0, Chicago, IL, USA). The microbial community abundance (%), according to different taxonomic levels, richness and diversity indices (ACE, Chao1 and Shannon index), were generated using the Mothur program. In heat map analysis, Spearman’s rank correlation coefficients were calculated using the R programming language (version 3.1.0) to decipher the relationships between abundant microbial taxa, soil edaphic factors and cucumber yield, as well as the relationships between key antagonists of Fusarium incidence inhibition. A Venn diagram was generated using R (Version 3.0.2) to elucidate the unique OTUs belonging to each treatment and the shared OTUs among the different treatments. Principal component analysis (PCA) was conducted on microbial community composition to show the sampling differences among treatments.

## 5. Conclusions

Short-term garlic substrate addition is capable of modifying soil biochemical properties by contributing to organic C input, providing idyllic niches in the food webs of microbiota and leading to the process of organic matter mineralization. Such factors subsequently accelerated biological activities, induced the microbial community diversity and thus improved soil–plant growth productivity. Garlic substrate increased the abundances of plant-beneficial microbes that significantly contributed to antagonizing pathogens and thus promoting crop yield. We suggest that garlic substrate amendment is ecologically sound and compatible with local natural and socio-economic structures. It may be an excellent option to restructure degraded soil micro-ecological environments over a short duration to improve plant growth, especially where the soil–plant–microbiome structure of PGVC crops is drastically degraded by the continuous cropping systems used across the world. Further environment-specific metabolomics studies through the quantitative modeling of microbiomes, however, are necessary to explore how garlic-mediated plant–microbe interactions could be useful in the environmentally stressed rhizosphere ecology of PGVC systems.

## Figures and Tables

**Table 1:** Effect of different concentrations of garlic substrate on plant growth, fruit yield and Fusarium wilt incidence rate.

Plant Growth Indexes	CK	T1 (1:100)	T2 (3:100)	T3 (5:100)
Plant height (cm)	75.16 ± 8.34 b	81.58 ± 3.33 b	88.27 ± 1.46 ab	94.53 ± 4.23 a
Leaf area (cm^−2^)	163.05 ± 7.48 b	166.82 ± 3.73 b	177.65 ± 3.39 b	219.91 ± 4.69 a
Shoot fresh weight (g/plant)	75.84 ± 10.92 b	72.51 ± 8.27 b	91.79 ± 3.44 ab	107.85 ± 4.64 a
Shoot dry weight (g/plant)	12.47 ± 2.90 b	10.94 ± 2.74 b	16.69 ± 0.99 a	17.21 ± 0.98 a
Root fresh weight (g/plant)	5.90 ± 0.65 bc	5.02 ± 0.32 c	8.97 ± 0.88 a	7.13 ± 0.31 ab
Root dry weight (g/plant)	0.86 ± 0.14 b	0.87 ± 0.06 b	1.87 ± 0.12 a	1.45 ± 0.23 a
Fruit fresh weight (g/plant)	171.45 ± 6.39 b	163.74 ± 5.49 b	213.98 ± 5.82 a	197.30 ± 9.41 a
Fruit length (cm)	31.60 ± 1.16 a	32.75 ± 1.43 a	34.74 ± 1.03 a	35.01 ± 1.53 a
Cucumber yield (g/plant)	725.12 ± 7.72 c	749.50 ± 16.76 c	822.8 ± 9.25 b	876.92 ± 6.00 a
Fusarium wilt incidence %	33.63 ± 0.52 c	30.12 ± 1.21 b	26.53 ± 2.52 ab	21.53 ± 1.32 a

Values within a column followed by the same letter(s) are not significantly different at *p* < 0.05, based on one-way analysis of variance with least significant difference test (LSD) test.

**Table 2:** Soil physicochemical and biological properties affected by different concentrations of garlic substrate.

Soil Properties	CK	T1 (1:100)	T2 (3:100)	T3 (5:100)
Soil pH (1:5 soil: Water)	7.75 ± 0.04 b	7.94 ± 0.01 a	7.80 ± 0.04 b	7.63 ± 0.02 c
EC (µs·cm^−1^)	588.56 ± 2.93 bc	581.12 ± 1.81 c	595.57 ± 0.11 b	611.59 ± 1.09 a
Organic matter (g·kg^−1^)	25.70 ± 2.93 c	28.16 ± 1.81 bc	31.94 ± 0.11 ab	35.89 ± 1.09 a
Available N (mg·kg^−1^)	75.16 ± 1.97 c	94.64 ± 2.15 b	107.83 ± 1.07 a	113.39 ± 1.46 a
Available P (mg·kg^−1^)	72.83 ± 3.07 c	119.26 ± 2.34 b	129.83 ± 1.09 a	136.61 ± 2.91 a
Available K (mg·kg^−1^)	333.18 ± 1.46 c	348 ± 1.60 b	355.83 ± 1.83 a	346.35 ± 3.31 b
Soil invertase (glucose mg·g^−1^ soil d^−1^)	27.3 ± 1.97 b	31.08 ± 1.47 b	32.64 ± 1.14 b	39.31 ± 1.94 a
Urease (NH_3_-N mg·g^−1^ soil d^−1^)	3.06 ± 0.23 c	4.27 ± 0.39 b	4.69 ± 0.44 b	6.71 ± 0.23 a
Catalase (KMnO_4_ mL·g^−1^ 20 min^−1^)	10.71 ± 0.64 b	11.08 ± 0.81 b	12.78 ± 0.23 ab	13.96 ± 0.97 a
Alkaline phosphatase (P_2_O_5_ mg 100 g^−1^ soil d^−1^)	1.56 ± 0.10 c	1.93 ± 0.26 bc	2.28 ± 0.10 b	3.35 ± 0.27 a

Values are means ± SE, *n* = 3. Mean values within columns followed by different letters are significantly different using the LSD test (*p* < 0.05).

**Table 3:** Comparison of the estimated OTUs richness and diversity indices of microbes clustering at 97% identity, as obtained from Illumina sequencing under different treatments. Taxonomic alpha diversity was measured as OTU richness (number of OTUs), estimated richness (Chao1 and ACE), and diversity (Shannon index).

Treat-Ments	Bacterial 16S rRNA				Fungal 18S rRNA			
	OTUs	ACE	Chao1	Shannon	OTUs	ACE	Chao1	Shannon
CK	2190 ± 24.36 ab	2601 ± 13.09 b	2612 ± 13.5 b	6.66 ± 0.05 ab	110.9 ± 7.24 bc	125 ± 7.0 b	124 ± 8.08 b	2.13 ± 0.23 ab
T1	2232 ± 23.48 ab	2625 ± 24.42 ab	2637 ± 23.11ab	6.59 ± 0.08 ab	141.3 ± 11.31 a	153.3 ± 8.37 a	155 ± 10.40 a	2.45 ± 0.38 ab
T2	2268 ± 47.92 a	2680 ± 21.36 a	2691 ± 16.55 a	6.98 ± 0.03 a	141.7 ± 6.43 a	150.3 ± 6.11 a	152.7 ± 7.35 a	2.89 ± 0.24 a
T3	2134 ± 26.74 b	2627 ± 17.98 ab	2605 ± 26.92 b	6.32 ± 0.43 b	128.7 ± 13.9 ab	151 ± 4.93 a	147 ± 7.0 ab	2.08 ± 0.19 b

Average of three replicates with standard error (*n* = 3, mean ± SE) of each treatment. CK (control), T1 (1:100), T2 (3:100), T3 (5:100).

## Supplementary Materials

Supplementary Materials can be found at https://www.mdpi.com/1422-0067/21/17/6008/s1.
