# Supplementary material for: Garlic Substrate Induces Cucumber Growth Development and Decreases Fusarium Wilt through Regulation of Soil Microbial Community Structure and Diversity in Replanted Disturbed Soil
Source: Int J Mol Sci. 2020 Aug 20;21(17):6008. doi: 10.3390/ijms21176008 (PMC7504009; doi:10.3390/ijms21176008)
Supplement: Supplementary file 1 [file ijms-21-06008-s001.pdf]

Garlic Substrate Induces Cucumber Growth Development and Decreases Fusarium Wilt through Regulation of Soil Microbial Community Structure and Diversity in Replanted Disturbed Soil

Ahmad Ali [a,b], Muhammad Imran Ghani [a], Haiyan Ding [a], Muhammad Iqbal [c], Zhihui Cheng[a,]*, Zucong Cai [b,d,e]

[a] *College of Horticulture, Northwest A&F University, Yangling, Shaanxi 712100, China.*

[b] *School of Geography Science, Nanjing Normal University, Nanjing 210023, China*

[c] *Institute of Soil Science, PMAS-Arid Agriculture University, Rawalpindi-46300, Pakistan..*

[d] *State Key Laboratory Cultivation Base of Geographical Environment Evolution 14, Nanjing 210023, China*

[e] *Jiangsu Provincial Key Laboratory of Materials Cycling and Pollution Control, Nanjing Normal University, Nanjing 210023, China*

*Corresponding Author: Zhihui Cheng

E-mail: chengzh@nwsuaf.edu.cn

Phone number: +86 29 87091332

Fax number: +86 29 87082613

Supporting Information

3 Tables

2 Figure

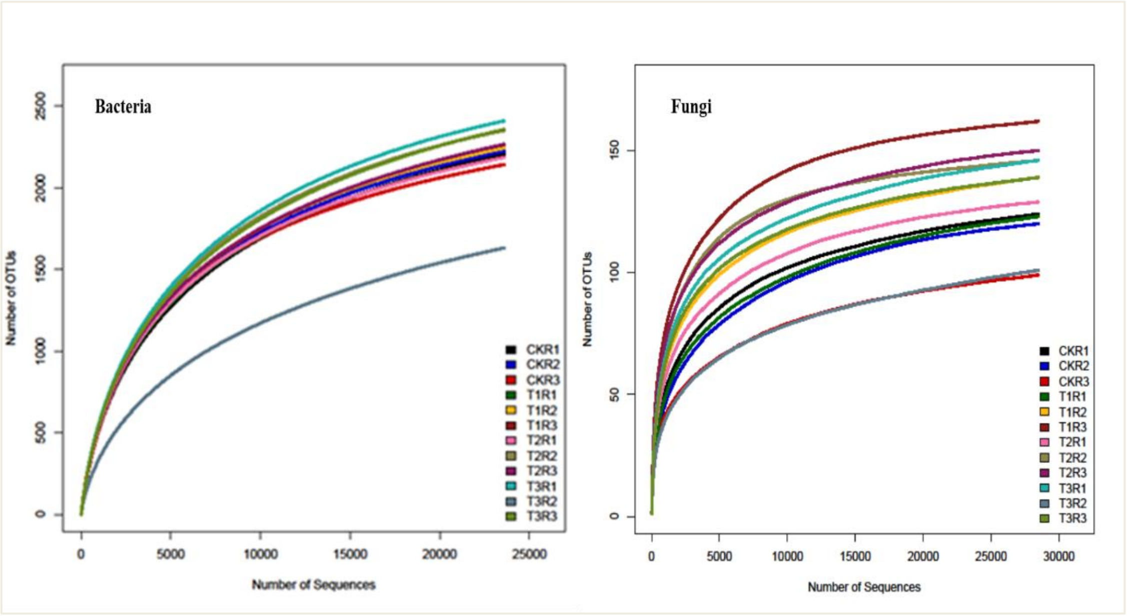

Figure S1. Rarefaction curves of bacterial and fungal communities based on observed OTUs at 3% distance associated with different soil samples.

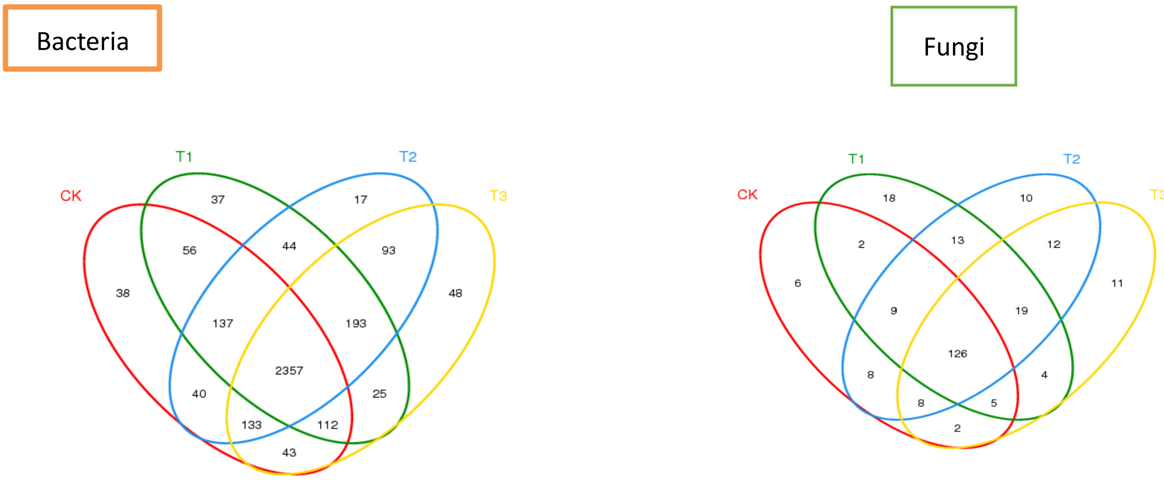

Figure S2. A Venn diagram displaying the degree of overlap of bacterial OTUs (a) and fungal (b) at the 3% evolutionary distance among the four treatments.

Calculations based description of Venn diagram based on OTUs in term of uniqueness and shareness among different soil samples.

	**Bacteria**				**Fungi**	
	**Total OUT in individual samples**	**Unique OTUs**	**Shared OTUs (b/w treatments)**		Unique OTUs	Shared OTUs(b/w treatments)
**Total OTUs identified in 4 treatment samples: 2918,ck)+(2961,T1)+(3014,T2)+(3004,T3) =11897**	CK:2918 (24.52%)	CK: 38 (1.30%)	CK-T1: 56+137+2357+112=2662	**Total OTUs identified in 4 treatment samples:**	CK: 6	CK-T1: 5+126+9+2=152
	T1:2961 (24.88%)	T1: 37 (1.24%)	CK-T2:137+40+133+2357=2667		**T1: 18**	CK-T2: 9+126+8+8=151
	**T2: 3014 (25.33%)**	T2: 17 (0.56%)	CKT3: 2357+133+43+112=2645		T2: 10	CKT3:126+5+8+2=141
	T3:3004 (25.25%)	**T3: 48 (1.59%)**	T1-T2: 44+137+193+2357=2731		T3: 11	T1-T2:13+9+126+19=**167**
			T1-T3: 2357+112+25= 2494			T1-T3:19+4+5+126=154
			T2-T3: 93+193+2357+133=**2776***			T2-T3:12+19+126+8=165
			Overall total OTUs in sheerness: 2662+2667+2645+2731+ 2494 + 2776=15975		Overall total unique OTUs: 6+18+10+11=45	Overall total OTUs in sheerness: 152+151+141+167+ 154 + 165=930
		**Remarks:**				
		Highest Unique. **T3:48**	Max shared OTUs: **T2-T3 (2776)**		Highest Unique. **T1:18**	Max shared OTUs: **T1-T2 (167)**

Table S1. Pearson correlations between crop yields, Fusarium wilt incidence rate % and primary environmental parameters.

Pearson's correlation between crop yield Fusarium wilt incidence rate % and soil properties									
	**CY**	**pH**	**OM**	**AN**	**AP**	**AK**	**Inv.**	**UR**	Cat.
pH	-.634								
OM	**.995****	-.589							
AN	.780	-.012	**.974***						
AP	**.975***	-.152	.888	**.988***					
AK	.566	.235	.580	.902	.833				
Inv.	**.979***	-.565	**.973***	**.987***	.874	.478			
UR.	.945	-.550	**.970***	**.994****	.879	.485	**.996****		
FWI.	**-.961***	-.784	**-.953***	-.868	-.847	**-.972***	-.879	-.870	
A. POH.	**.959***	-.656	**.973***	.724	**.982****	.409	**.993****	**.991****	.915

*. Correlation is significant at the 0.05 level (2-tailed).
**. Correlation is significant at the 0.01 level (2-tailed).

CY: Crop Yield; **FWI:** Fusarium wilt incidence rate %; **EC:** Electrical Conductivity; **OM:** Organic Matter; **AN:** Available Nitrogen; **AK:** Available Potassium; **Inv:** Invertase; **UR:** Urease; **A. POH**: Alkaline phosphatase

Table S2. Correlation analysis between cucumber yield, Fusarium incidence% and microbial richness and diversity indices.

		Cucumber Yield	Fusarium incidence%
**Bacteria**	OTUs	**.978***	-.861
	ACE	**.960***	-.771
	Chao1	.911	**-.957***
	Shannon	**.973***	**-.974***
**Fungi**	OTUs	.888	-.864
	ACE	.853	-.752
	Chao1	.615	.864
	Shannon	.854	**-.971***

*Correlation is significant at the 0.05 level (2-tailed).

**Correlation is significant at the 0.01 level (2-tailed).

Alpha diversity indices of community richness index (OTU, Chao1, and ACE)

Community diversity index (Shannon)

OTU: Observed species

Table S3. Basic characteristics of replanted soil and garlic substrate before experiment

Parameters	Replanted soil	Garlic substrate
Soil pH (1:5 soil: water)	7.75	7.25
EC (μs cm^{-1})	582	671
Total organic C (g kg^{-1})	13.59	411.39
Total N (g kg^{-1})	1.438	8.43
C:N	9.45	49
Total P (g kg^{-1})	0.93	18.74
Total K (g kg^{-1})	7.15	10.27
Available N (mg kg^{-1})	53.65	-
Available P (mg kg^{-1})	59.41	-
Available K (mg kg^{-1})	305.91	-
